# A Multi-Site Cross-Sectional Study of Anxiety Symptoms and the Associated Factors Among Chinese Drug Users Undergoing Compulsory Detoxification Treatment

**DOI:** 10.3389/fpubh.2021.524068

**Published:** 2021-03-11

**Authors:** Xiaoshi Yang, Carrie Kovarik, Yuke Wang, Shenshui Yu

**Affiliations:** ^1^Department of Social Medicine, School of Public Health, China Medical University, Shenyang, China; ^2^Department of Dermatology, University of Pennsylvania, Philadelphia, PA, United States; ^3^Liaoning Juvenile's Compulsory Drug Detention Center, Shenyang, China

**Keywords:** anxiety symptoms, perceived stress, optimism, drug users, compulsory detoxification treatment

## Abstract

Compulsory drug detoxification treatment (CDT) is currently the major drug rehabilitation modality in China, and drug users often suffer from extraordinary levels of stress during CDT, leading to a high prevalence of anxiety symptoms. This study assesses anxiety symptoms of the drug users undergoing CDT and explores the associated factors. A cross-sectional study with cluster sampling was conducted in three cities in Liaoning Province of Northeast China. Nine hundred CDT drug users were interviewed face-to-face with Chinese questionnaires. Hierarchical multiple regression (HMR) analysis was conducted to explore the factors associated with anxiety symptoms. The prevalence of anxiety symptoms among the CDT drug users was substantially high (33.2%). HMR analysis indicated perceived stress and characteristics of drug use such as types of drugs, were the most important contributors to anxiety symptoms. Optimism (LOT-R) played a protective role in reducing anxiety symptoms in this population. Anxiety symptoms of drug users undergoing CDT were present in a significant proportion of the CDT population. Optimism is a protective factor which could attenuate the detrimental effects of perceived stress on anxiety symptoms and potentially improve treatment outcomes.

## Introduction

Illicit drug use has spread dramatically in China since the 1980's, becoming a critical public health problem. According to China's National Narcotics Control Commission 2016 Annual Report on Drug Control in China, synthetic drugs such as methamphetamine and other amphetamine-type stimulants have surpassed heroin to become the most abused drugs in China. Abuse of various new psychoactive substances, such as ketamine, is also increasingly prevalent. By the end of 2014, the number of registered drug users in China reached 2.96 million (compared to 70,000 in 1990) (1).

Drug addiction impacts the individual user, their families, and society, with loss productivity and revenue, engagement in high risk behaviors, transmission of communicable diseases, increase in criminal behavior, increase in health care expenditures, and stress on interpersonal relationships. Given the exponential rise in drug use over the past several decades and the impact on a significant population in China, the 2008 Anti-Drug Law of China created a new drug detoxification system. Compulsory drug detoxification treatment (CDT) is currently the major drug rehabilitation modality in China (2). Drug addicts who have refused community based rehabilitation, have failed to maintain abstinence, or have been arrested for having a severe drug addiction are sent to this 1–3 year program, which is managed by the Ministry of Justice (2, 3). The CDT, which restricts drug users' daily lives and freedom, is different from that in Western countries. It is a compulsory administrative measure and is issued by the public security bureau and enforced by the judicial administrative bureau.

The drug users on CDT are a vulnerable population, exposed to numerous inner conflicts, feelings of isolation, lack of support, and hardships of daily life. The struggles and stressful experiences due to this treatment not only bring about psychological and physical distress, but also result in the high levels of perceived stress, which is the degree to which situations in one's life are appraised as stressful. In addition, the drug users are more susceptible to negative feelings, such as helplessness and hopelessness, or feelings of guilt in adverse situations which could contribute to future relapse. Previous research has reported that drug users have an increased prevalence and comorbidity of psychiatric disorders, such as depression and anxiety as compared to the general populations, which also need to be addressed during treatment (4–6).

Psychiatric comorbidity is a major issue that should be addressed when treating patients with substance abuse in order to maintain abstinence. A recent study by Dong et al. from both compulsory and voluntary treatment centers in China shows significant differences in the psychiatric comorbidity, based on the drug of choice, with methamphetamine-dependence related to higher rates of psychotic disorders and heroin-dependence related to higher rates of substance-induced mood disorders (7, 8). Previous research by Yin et al. in one of China's methadone maintenance treatment centers indicated a high prevalence of anxiety among drug users in treatment (18.4 %) and a significant number with co-occurring depression (14.2%) (9).

The transactional stress model theory proposed by Lazarus and Folkman (10) can be applied to explain how major life events and daily challenges impact mental health. This process is influenced by cognitive appraisal, including assessing threats, and coping with stress (11–14). The commonly used transactional stress model indicated that cognitive appraisal could affect the process of life events and coping styles when tacking with difficulties, and made significant contribution to the individual's mental health among the drug users. CDT is a major life event for the drug user and brings about tremendous challenges in their daily life and psychological reaction, which may have an detrimental impact on mental and emotional well-being, placing them at risk for psychological disorders. Moreover, perceived stress can result in physiologic or psychological impairment that puts them at risk for anxiety. Based on previous studies (15), higher levels of perceived stress negatively affect drug users' psychological health, often correlating with symptoms of anxiety and depression (16–18).

However, the adoption of positive psychological capabilities has been found to be inversely related to anxiety among drug users (19). The positive capabilities, such as having an optimistic attitude, appear to have positive effects on the psychological well-being and could attenuate the prevalence of anxiety symptoms. Optimism is a positive psychological resource with a tendency to expect favorable outcomes about the future (20). It has been found to be associated with more active coping strategies, lower levels of psychological distress, health-enhancing behavior, and improved outcomes in drug rehabilitation. In one study, the extent of professed optimism about one's future distinguished between those showing positive and negative outcomes in drug rehabilitation. In addition, optimism and pessimism were the only psychological measure to serve as a predictor of post-treatment drug use (21). However, studies evaluating the influences of positive psychological resources, such as optimism, on drug users in treatment are underrepresented, and relevant studies have not been conducted among the CDT drug users in China.

Despite the availability of health care services, there are still barriers to address the psychological needs of CDT drug users in China. This study, therefore, evaluates the prevalence of anxiety symptoms experienced by drug users who are in CDT and explore the predictors of anxiety symptoms. The findings from this study will give effective directions to deliver necessary health care services in order to improve mental health and psychological well-being among drug users undergoing CDT.

## Methods

### Sample and Data Collection

A cross-sectional study with clustering sampling was conducted in three cities in Northeast China (Shenyang, Dalian and Yingkou) from February 2016 to October 2016. Eligible subjects were those undergoing CDT in rehabilitation, at least 18 years old and without diagnosed severe independent psychiatry diseases, including anxiety and panic disorders, bipolar disorder, depression, eating disorders, and schizophrenia. This study was collaborated with Liaoning Juveniles Compulsory Drug Detention and Detoxification Center and Centers for Disease Prevention and Control (CDC) of Dalian and Yingkou. A total of 900 CDT drug users who were screened for eligibility and met the inclusion criteria were enrolled in this study. Trained correctional officers conducted face-to-face interviews using the study questionnaires at the project office with the 793 CDT participants who completed the study. The characteristics (age, sex, characteristics of drug use and rehabilitation) of the included participants were similar to those of the 107 CDT drug user who were excluded.

### Ethics Statement

Written informed consent was obtained prior to enrollment from each participant. Face-to-face interviews lasting for ~20–30 min were conducted by trained surveyors at the project office where privacy was well-protected. The procedures conformed to ethical standards and approved by the Committee on Human Experimentation of China Medical University.

### Measures

#### Assessment of Anxiety Symptoms

Anxiety symptoms were measured with the Zung Self-Rating Anxiety Scale (SAS) (22), which is composed of 20 items with four possible responses: (1) never, (2) rarely/sometimes, (3) frequently, and (4) always. Each item was scored on a 4-point Likert according to severity of anxiety disorder. The raw score was standardized according to the formula: standard score = int (1.25^*^raw score). According to the Chinese norm, when a score exceeded 50, it was determined that the person suffered from anxiety symptoms (23). Higher scores indicate greater perceived anxiety symptoms.

#### Demographic Characteristics of the Participants

Demographic characteristics included age, marital status (never married, married or others which included widowed, divorced or separated), sex, level of education, and monthly income. Level of education was classified as “primary school or below,” “junior high school,” or “senior high school or above.” Monthly income was categorized as “ ≤ 2,000 yuan,” “2,001–3,000 yuan,” “3,001–5,000 yuan,” or “≥50,001yuan.”

#### Characteristics of Drug Use

Characteristics of drug use consisted of types of drugs, method of drug delivery, provision source of drugs, frequency of drug use, and duration of drug use. Types of drugs was categorized as “opioids,” including heroin, morphine and others, “synthetic,” such as ice and ecstasy or “combination.” Method of drug delivery was divided into “mouth and nose inhalation,” “intravenous drug use,” and “other methods.” Provision source of the drugs included “provided from partners/friends,” “provided from drug dealers,” and “both sources.” Frequency of drug use was classified as “more than once a day~ once/day,” “once a week~ once every other week,” and “once a month ~ occasionally.” Duration of drug use was categorized as “ <1year,” “1-2 years,” “2-5 years,” or “≥5 years.”

#### Characteristics of Rehabilitation of CDT

Characteristics of the current rehabilitation of CDT included degree of willingness of drug abstention, number of times of CDT, and period of time of current CDT (how long they had been in the current period of CDT). Degree of willingness of drug abstention was classified as “extreme,” “slight,” and “none.” Number of times of CDT was categorized as “one time,” “two times,” and “three times or more.” Period of the current CDT was categorized as “ <3 months,” “3–12 months,” “12-18 months,” and “≥18 months.”

#### Measurement of Perceived Stress

The Perceived Stress Scale (PSS) was employed to assess the perceived stress (24), which consisted of ten items that involved questions measuring the individual's feelings and thoughts in last month. The responses for each item ranged from 0 (never) to 5 (very often). Answers were summarized to create an overall score ranging from 0 to 40, with higher scores indicating the higher levels of perceived stress. The Cronbach's alpha coefficient for this scale in this study was 0.949.

#### Measurement of Optimism

Optimism was assessed with the Life Orientation Test Revised (LOT-R) (25), which was devised to measure individual differences in generalized optimism vs. pessimism and had been validated and widely used in many countries, including United States, Brazil, Germany, Norway and China (26). The LOT-R consisted of 10 items, which were rated on a 5-point Likert scale ranging from 1 (I strongly agree) to 5 (I strongly disagree), while the items reflecting pessimism were scored from 5 to 1. Total scores were calculated by summing the three positively worded items and three negatively worded items that were reversely coded, ranging from 6 to 30, with lower scores indicating higher levels of optimism. The Cronbach's alpha coefficient was 0.707 in this study.

### Statistical Analysis

The distributions of SAS scores among the categorical variables were depicted by *T*-tests and one-way ANOVA. Pearson's correlation was used to assess the correlations between SAS scores and the related factors. All the continuous variables were standardized in order to avoid multicollinearity before performing the regression analysis (27). Hierarchical multiple regression (HMR) analysis was employed to test the incremental variance by using a set of independent variables. The SAS scores were used as dependent variables, with the independent variables being entered in the following steps: Step 1: demographic characteristics of the drug user; Step 2: characteristics of drug use; Step 3: characteristics of current rehabilitation; Step 4: perceived stress and Step 5: optimism. Standardized parameter estimates (the standardized β) were used in order to make comparisons of the magnitudes of the associations across independent variables. The fit of the model was assessed with the *R*^2^-value. Statistical analyses were conducted using SPSS 17.0, and a two-tailed probability value of <0.05 was considered to indicate statistical significance.

## Results

### Description of the Participants

The valid response rate was 88.11% in this study. The basic characteristics of CDT drug users are provided in Table 1. The participants' average age was 33.34 ± 8.86 years old (ranging from 18 to 63 years old). Most (60.2%) of the participants had a maximum education level of junior high school. There were 410 drug users addicted to synthetic drugs (51.7%), 138 addicted to opioid type drugs (17.4%), and 245 drug users (30.9%) addicted to both types of drugs. The majority of drugs were delivered through mouth and nose inhalation (61.7%). The duration of drug use varied, but a slight majority had been using for 2 years or less (56.2%).

**Table 1:** Baseline characteristics of study participants and distribution of anxiety symptoms (*n* = 793).

	**n**	**%**	**SAS**
**Age**			
<30	328	41.4	43.99 ± 10.962
30–35	175	22.1	44.32 ± 13.010
>35	290	36.5	47.19 ± 11.446^**^
**Sex**			
Men	373	47	45.00 ± 11.937
Women	420	53	45.44 ± 11.492
**Marital status**			
Never married	337	42.5	44.84 ± 11.748
Married	259	32.7	44.55 ± 10.660
Other	197	24.8	46.8 ± 12.785
**Levels of education**			
Primary school or below	128	16.1	50.14 ± 10.762^**^
Junior high school	477	60.2	44.12 ± 11.268
Senior high school or above	188	23.7	44.72 ± 12.563
**Monthly income (yuan)**			
<2,000	240	30.3	45.48 ± 12.031
2,001–3,000	261	32.9	43.40 ± 12.858
3,001–5,000	187	23.6	46.75 ± 9.982^*^
≥5,001	105	13.2	46.54 ± 10.176^*^
**Types of drugs**			
Synthetic drugs	410	51.7	47.79 ± 11.148^**^
Opioids	138	17.4	37.36 ± 9.747
Combination	245	30.9	45.39 ± 11.698^*^
**Methodof drug delivery**			
Mouth and nose inhalation	489	61.7	47.20 ± 11.460^**^
Intravenous drug use	151	19.0	40.85 ± 11.774
Other methods	153	19.3	43.28 ± 10.923^*^
**Duration of drug use**			
<1year	231	29.1	39.53 ± 11.291
1-2 years	215	27.1	47.67 ± 11.134^*^
2-5 years	196	24.7	46.27 ± 10.213^*^
≥5 years	151	19.0	49.14 ± 11.803^**^
**Frequency of drug use**			
More than once a day-once/day	334	42.1	48.54 ± 11.351^**^
Once a week-once every other week	190	24.0	46.14 ± 10.491^*^
Once a month-occasionally	269	33.9	40.48 ± 11.392
**Provision source of the drugs**			
Provided from Drug dealers	160	20.18	49.55 ± 11.00
Provided from Partners/Friends	145	18.28	46.97 ± 9.70
Both sources	349	44.01	40.91 ± 11.79^**^
Other sources	139	17.53	49.29 ± 10.42
**Period of time of current CDT**			
<3 months	299	37.7	39.88 ± 10.951
3-12 months	170	21.4	49.00 ± 11.365^**^
12-18 months	171	21.6	49.06 ± 11.233^**^
18 months	153	19.3	47.24 ± 10.022^**^
**Number of times of CDT**			
One time	500	63.1	43.91 ± 12.263
Two times	166	20.9	47.30 ± 10.521^**^
Three times or more	127	16	47.74 ± 10.035^**^
**Willingness of drug abstention**			
Extreme	442	55.7	42.20 ± 11.206
Slight	228	28.8	48.23 ± 11.365^*^
None	123	15.5	50.59 ± 10.723^**^

* *Significant at the 0.05 level (two-tailed)*;

** *Significant at the 0.01 level (two-tailed)*.

*CDT, compulsory detoxification treatment*.

More than half of the drug users expressed extreme willingness to recover from drug addiction, while 28.8 and 15.5% of the drug users had slight to no willingness. Almost 37% of the drug users had received CDT more than once. Most participants in current CDT had been present for <3 months (37.7%), while only a small proportion had been there more than 18 months (19.3%).

### Anxiety Symptoms of the CDT Drug Users

Table 1 shows the distributions of anxiety symptoms among participants. The prevalence of anxiety symptoms among drug users undergoing CDT was 33.2% (SAS≥50). The study found that drug users 35 years and older, with only a primary school education, addicted for more than 5 years, used drugs at least once per day, and used synthetic drugs had greater perceived anxiety symptoms. There was a significant difference between the anxiety symptoms of mouth and nose inhalation and intravenous drug use (*P* < 0.01), with those preferring inhalation methods reporting greater perceived anxiety symptoms. Drug users whose current CDT has lasted longer than 3 months exerted greater SAS anxiety scores than those who had been present for <3 months. The participants with slight to no willingness of drug abstention reported higher anxiety symptoms than those with significant willingness of drug abstention. The participants who had been to CDT two or more times had significantly (*P* < 0.01) more anxiety than those that were in CDT for the first time.

### Correlation of Anxiety Symptoms and Related Factors

Table 2 shows the results of the correlation analyses between SAS scores and continuous variables. Age and perceived stress were significantly and positively correlated with SAS anxiety scores (*P* < 0.01). With respect to positive psychological capabilities, lower levels of optimism in participants (higher the LOT-R scores) correlated with higher the anxiety symptoms (*P* < 0.01).

**Table 2:** Correlation of SAS and related factors.

	**M**	**SD**	**1**	**2**	**3**	**4**	**5**
1. Anxiety symptoms (SAS)	45.23	11.698	1				
2. Age	33.35	8.862	0.134^**^	1			
3. Social support	61.50	15.148	−0.169^**^	−0.116^**^	1		
4. Perceived stress	15.92	5.535	0.531^**^	0.059	−0.228^**^	1	
5. Optimism (LOT-R)	15.27	3.432	0.438^**^	0.025	−0.264^**^	0.507^**^	1

** *Significant at the 0.01 level (two-tailed); The higher LOT-R score indicating the low levels of optimism*.

### Hierarchical Multivariate Regression Predicting Anxiety Symptoms

The HMR models of SAS score are shown in Table 3. Each block of the independent variables made a significant contribution to the variance of anxiety symptoms (*P* < 0.05). A total of 42.2% of variance was explained by the final regression model in anxiety symptoms. The *R*^2^ changes indicated the incremental variance explained by the demographics characteristics, characteristics of drug use, characteristics of current rehabilitation, perceived stress and optimistic of variables were 5.7, 18.3, 1.8, 15.5, and 0.9%, respectively. It was also indicated that the characteristics of drug use and perceived stress made the most significant contributions to the variance of anxiety symptoms.

**Table 3:** The hierarchical multiple regression models of anxiety symptoms.

	**Anxiety symptoms**				
**Variables**	**Model 1 (Beta)**	**Model 2 (Beta)**	**Model 3 (Beta)**	**Model 4 (Beta)**	**Model 5 (Beta)**
**Block 1 Demographic characteristics**					
**Age**	0.148^**^	0.118^**^	0.116^**^	0.103^**^	0.106^**^
**Marital status**					
Other vs. Married	0.060	−0.035	−0.046	−0.049	−0.048
Never married vs. Married	0.078	−0.005	−0.018	−0.035	−0.032
**Sex**	0.049	−0.043	−0.030	−0.015	−0.017
**Levels of education**					
Primary school vs. Junior high school	0.190^**^	0.149^**^	0.153^**^	0.131^**^	0.128^**^
Senior vs. Junior high school	0.019	−0.011	−0.013	0.016	0.020
**Monthly income**	0.035	−0.016	−0.022	−0.031	−0.029
**Block 2 Characteristics of drug use**					
**Types of drugs**					
Synthetic vs. Opioids		0.166^**^	0.138^*^	0.117^*^	0.102^*^
Combination vs. Opioids type		0.268^**^	0.238^**^	0.207^**^	0.191^**^
**Method of drug delivery**					
Mouth and nose inhalation vs. Intravenous drug use		0.026	0.001	0.006	0.004
Other methods vs. Intravenous drug use		−0.009	−0.006	−0.034	−0.033
**Provision source of the drugs**					
Provided from partners/friends vs. drug dealers		−0.042	−0.035	−0.025	−0.033
Both sources vs. Provided from drug dealers		−0.199^**^	−0.170^**^	−0.075	−0.063
Other sources vs. Provided from drug dealers		0.028	0.018	0.030	0.019
**Frequency of drug use**		−0.130^**^	−0.092^*^	−0.041	−0.033
**Duration of drug use**		0.106^**^	0.063	0.049	0.040
**Block 3 Characteristics of current rehabilitation**					
Willingness of drug abstention					
Slight vs. extreme			0.071^*^	0.046	0.046
None vs. extreme			0.110^**^	0.069^*^	0.072^*^
Number of times of CDT			0.033	−0.008	−0.015
Period of time of current CDT			0.084^*^	0.099^**^	0.092^*^
**Block 4 Perceived stress**				0.425^**^	0.377^**^
**Block5Optimism**					0.120^**^
R^2^	0.057	0.240	0.258	0.413	0.422
ΔR^2^	0.057	0.183	0.018	0.155	0.009

* *Significant at the 0.05 level (two-tailed)*;

** *Significant at the 0.01 level (two-tailed)*.

*CDT, compulsory detoxification treatment*.

The final HMR models revealed that age, educational level, types of drugs, willingness of drug abstention, period of time of current CDT, perceived stress, and optimism (LOR-T) were significant predictors of anxiety symptoms. The anxiety symptoms were significantly and positively associated with the age, period of current CDT, and perceived stress, yet significantly and inversely associated with optimism (*P* < 0.01). The study showed that perceived stress was the second highest contributor to anxiety symptoms (15.5% of variation in the model being explained), and it was the most important associated factor for anxiety symptoms. The greater the perceived stress, the greater the anxiety symptoms were; however, optimism (with lower LOR-T score) correlated with a lower perceived anxiety and may play a role in attenuating anxiety symptoms.

## Discussion

The results from this study indicated that anxiety symptoms were severe among the drug users undergoing CDT, with a prevalence of anxiety symptoms of 33.2%, which is significantly higher than those of the general public (ranging from 13.6 to 28.8%) (28, 29) and the patients attending methadone maintenance treatment in China (18.4–19.5%) (9, 30). However, it was lower than reported levels of anxiety in Hispanic drug users in Puerto Rico (31), Brazilian LSD users (64.32%) (32) and Chinese drug users with CDT in Yunnan (SAS = 52.32) (33). Age, levels of education, types of drugs, willingness of drug abstention, and perceived stress were predictors of anxiety symptoms. Furthermore, optimism may have played a positive role in attenuating the anxiety symptoms brought by CDT.

The characteristics of drug use were of the greatest importance in predicting anxiety symptoms, accounting for 18.3% of the total variance. The drug users who used synthetic drugs were more likely to suffer from anxiety symptoms, which were different results from a previous study that showed no significant differences in anxiety symptoms between drug users who used synthetic drugs and opioids (34). Given the shifting preference for synthetic drugs in China, as also seen in this study, it is important to realize the changing psychiatric comorbidities that now may be seen in drug users undergoing CDT (35, 36). These changes may impact the approach taken in CDT, as well as the availability of mental health services to best help drug users through their journey to maintain abstinence.

In terms of characteristics of rehabilitation, willingness of drug abstention was a predictor of anxiety symptoms. Drug users who were extremely willing to abstain from drugs suffered from lower levels of anxiety symptoms than those with slight or no willingness. Personal subjective willingness and motivation may be essential for the successful treatment during the process of rehabilitation. The drug users who were extremely willing may actively cope with treatment through positive psychological capabilities, such as optimism, and take advantage of available resources, therefore, feeling less stressed and anxious. On the other hand, those that are much less willing to abstain from drugs are more prone to anxiety, and also have many of the risk factors for relapse after treatment, such as excessive dependence on drugs, lack of social support, relapse after several attempts of rehabilitation, or dissatisfaction with life after rehabilitation (37).

Perceived stress was the next most important predictor of anxiety symptoms. The results showed that the greater the perceived stress, the higher the prevalence of anxiety symptoms. CDT drug users generally perceive some degree of psychological stress, likely due to the loss of support, discrimination, and/or exclusion from their family members and society, as noted in a previous study (16). In addition, the environment of CDT itself may result in significant stress, given drug users are required or forced to engage in various activities and rehabilitation, which may or may not be desired by the individual. The drug users also have psychological barriers of returning to the society. They may worry about their failure of drug abstention, be self-accusatory, feel guilty, and have difficulties in controlling their future lives, which could lead to their perceiving stress during CDT rehabilitation. Some may have experienced several attempts at drug abstention before returning to CDT, which compounds their vulnerability, feeling of pressure to succeed, or loss in confidence; therefore, leading to additional anxiety symptoms, as was shown in our population (38).

Optimism played a protective role in reducing anxiety symptoms. The more optimistic the drug users were, the less likely they were to have symptoms of anxiety. A study by Shen et al. in a CDT in China showed that a negative mood correlated with cravings for methamphetamine (39). It was obvious that interventions on optimism enhancement for drug users could help them reduce the prevalence of anxiety symptoms. Given that optimism has been shown to decrease cravings, improve willingness for abstinence, and improve long term outcomes, incorporation of mental health services in CDT that allow drug users to improve positive psychological capabilities would be helpful (20).

## Limitations

This study exerts the limitation of being a cross-sectional design. Thus, conclusions about the causality between characteristics of drug and anxiety symptoms could not be derived. The face-to-face interview method is used to ensure the authenticity of the data; however, we are reliant on the quality of the recorded data. Besides, our study only assessed the related risk factors include drug users' demographic characteristics and characteristics of drug use, social support, stress and optimism without control group. The clinical information and unmeasured confounders need further study. However, the participants were selected with clustering sampling in three cities and the sampling size was large, which may allow for the generalizability to other populations.

## Conclusion

The prevalence of anxiety symptoms among drug users undergoing CDT is high. Anxiety symptoms are effectively predicted by perceived stress of the drug user and types of drugs they have been using. Moreover, optimism was an important protective factor for attenuating anxiety symptoms which stemmed from CDT. Providing psychological counseling for drug users in CDT which targets the following areas could effectively improve the prevalence of anxiety symptoms: (1) teach coping skills and the adoption of positive psychological capabilities, including optimism; (2) teach techniques to manage perceived stress; and (3) improve support programs for life after release from CDT.

## Data Availability Statement

The datasets generated for this study are available on request to the corresponding author.

## Ethics Statement

The studies involving human participants were reviewed and approved by the ethics committee of China Medical University. The patients/participants provided their written informed consent to participate in this study.

## Author Contributions

XY designed the study and provided input on the original research plan, analysis and interpretation of data and wrote the first draft of the manuscript. YW and SY contributed to the acquisition of subjects and conduction of the survey. CK contributed to the revision the manuscript. All authors have contributed to and approved the final manuscript.

## Conflict of Interest

The authors declare that the research was conducted in the absence of any commercial or financial relationships that could be construed as a potential conflict of interest.
